# Laparotomy, Remedy for Intussusception

**Published:** 1874-08

**Authors:** John Ashhurst


*AMERICAN JOURNAL OF THE MEDICAL SCIENCES—July*, 1874:

LAPAROTOMY, OR ABDOMINAL SECTION, AS A REMEDY FOR INTUSSUSCEPTION.—*By John Ashhurst, Jr., M.D.*—* * * From a study of the cases referred to in the preceding pages, the following conclusions may, I think, be considered as fairly established.

I. Past experience gives no encouragement to operative interference in cases of intussusception occurring in infants less than one year old.

II. When the symptoms present, and particularly the existence of intestinal hemorrhage, render it probable that the closeness of the intussusception will lead to sloughing of the invaginated portion, no operation is advisable; for while under these circumstances an operation would almost surely fail, there is a fair hope that separation of the invaginated mass may lead to spontaneous recovery.

III. There may be, however, exceptional cases, in which, while there is no prospect of recovery through sloughing, bloodless remedies fail to give relief, and the patient is in danger of succumbing through exhaustion and long-continued suffering; under such circumstances, if the age and general condition of the patient do not forbid it, the question of operative interference may properly be considered.

IV. When an operation is determined upon, *laparotomy* should invariably be preferred to either *enterotomy or colotomy*; these, though suitable operations in cases of congenital occlusion and chronic obstruction of the bowels, are unsuited for cases of intussusception or other varieties of acute obstruction.

V. In cases of acute intestinal obstruction from other causes than intussusception, should milder measures fail to give relief in the course of three, or at the most four, days, laparotomy should be unhesitatingly recommended, and may, under such circumstances, be resorted to with a reasonable hope of success.

